# Cooperative Full-Duplex V2V-VLC in Rectilinear and Curved Roadway Scenarios

**DOI:** 10.3390/s20133734

**Published:** 2020-07-03

**Authors:** Diego J. Cuba-Zúñiga, Samuel B. Mafra, J. Ricardo Mejía-Salazar

**Affiliations:** National Institute of Telecommunications (Inatel), Santa Rita do Sapucaí, MG 37540-000, Brazil; diego.cuba@mtel.inatel.br (D.J.C.-Z.); samuelbmafra@inatel.br (S.B.M.)

**Keywords:** visible light communication (VLC), vehicle-to-vehicle (V2V), vehicle-to-infrastructure (V2I), bit error rate (BER), full-duplex communication

## Abstract

We study here the vehicle-to-vehicle (V2V) visible light communication (VLC) between two cars moving along different roadway scenarios: (i) a multiple-lane rectilinear roadway and (ii) a multiple-lane curvilinear roadway. Special emphasis was given to the implementation of full-duplex (FD) cooperative communication protocols to avoid communication disruption in the absence of a line-of-sight (LOS) channel. Importantly, we found that the cooperative FD V2V-VLC is promising for avoiding communication disruptions for cars traveling in realistic curvilinear roadways. Results in this work can be easily extended to the case of vehicle-to-infrastructure (V2I), which can also be promising in cases of low-car-density environments.

## 1. Introduction

Owing to the low-complexity, scalability, positioning capabilities, improved security, resistance against weather conditions and cost-effective implementation, visible light communication (VLC) has emerged as an ideal candidate for vehicular communication applications [1,2,3,4]. Particularly important are the vehicle-to-vehicle (V2V) and vehicle-to-infrastructure (V2I) transmissions of security messages, which help to reduce, alert and prevent accidents by up to 81% [4]. This later application is of major importance for future safe autonomous vehicle networks [1,5,6]. V2V-VLC can be easily implemented through light-emitting diode (LED)-based headlamps, nowadays equipped in most modern cars, for short-range optical wireless communications [4,7]. The line-of-sight (LOS) communication between the transmitter (LED) and receiver (usually a photodetector (PD) or an image sensor) constitutes a channel with considerably reduced possibility of packet collision and/or interference, which in turn improves the security by preventing information theft and interception techniques [8,9,10]. The highly directional V2V-VLC, with reduced field-of-view (FOV), also enables the accommodation of a great number of simultaneous communications, using the total bandwidth for each communication link, in comparison to its RF counterpart [11]. Moreover, the experimental measurements in [12] indicate that the VLC can be reliably used for distances up to around 45 m in real-world highway driving scenarios that, at the expense of reduced communication distances (∼150 m in theoretical analyses) when compared with RF (∼500 m), can also obviate the spectrum shortage in RF systems [11]. VLC is also sensitive to changes in the network, where cars can loss the LOS communication, and then the half-duplex (HD) and full-duplex (FD) cooperative communication protocols are suitable to maintain the source-destination communication [13,14,15]. Despite these advantages, there are two important limitations, derived from the LOS transmission, which must be beaten before the implementation of V2V-VLC becomes a reality. First, interference from nearby emitting vehicles, ambient light sources and communication disruption by obstacles along straight roadways. Second, communication disruption by improper alignment between the emitter (LED) and receiver (PD). Although there is a vast amount of literature addressing the first issue [12,16,17,18,19], the misalignment between the LED and PD axes has been only partially addressed. Though a hybrid VLC-RF approach can be implemented [20], it can be costly and technically complex. Therefore, more research in VLC is needed to properly address this drawback. In particular, previous literature is mostly limited to misalignment effects on the communication performance for cars communicating between two different lanes along a straight highway [3,4,12,13,21].

The success rate of a V2V-VLC is influenced by many factors such as the attenuation, interference, noise and solar irradiance. All these effects have been extensively investigated in the available literature [12,16,17,19,22,23]. The signal attenuation, for example, is modeled in the VLC channel, whereas the sunlight and external light sources are considered shot-noise effects [24,25]. The presence of interfering nearby vehicles, on the other hand, has been recently shown to diminish the allowed source-destination separation [15,18]. Inspired by these latter works, we study here a more realistic scenario where the destination vehicle enters into a curved highway section, whereas the source vehicle continues emitting in the rectilinear section, which has received less attention in the literature. The system consists of a dual-hop cooperative network with an intermediary relay operating in a FD mode, based on the decode-and-forward protocol, because it outperforms the half-duplex mode in terms of throughput, as demonstrated in [15]. For a decode-and-forward process, the transmission occurs in two phases. At first, there exists a broadcast phase (BP), where the source (S) broadcasts its information; then, in the cooperative phase (CP), the relay (R) retransmits the message to the destination (D) [26]. The FD relaying has the potential to improve spectral efficiency, through simultaneous reception and transmission at the relay, in contrast to HD, where additional time slots are needed. The V2V-VLC performance is analyzed in terms of the bit error rate (BER) for two different geometrical configurations. For completeness purposes, we first study the system as an ad-hoc V2V-VLC network moving along a multiple-lane straight roadway. Second, the geometrical and VLC analyses are extended to the case when the straight roadway ends with a semicircular section. Results in this work indicate that cooperative communication protocols must be implemented as a way to avoid communication disruption when moving along realistic curved roadway scenarios.

This manuscript is organized as follows. The system model is presented in Section 2. The mathematical analysis of the FD V2V-VLC cooperative scheme is presented in Section 3. Numerical results are given in Section 4. Finally, conclusions are presented in Section 5.

## 2. System Model

### 2.1. Straight Roadway Scenario

**Figure 1:**
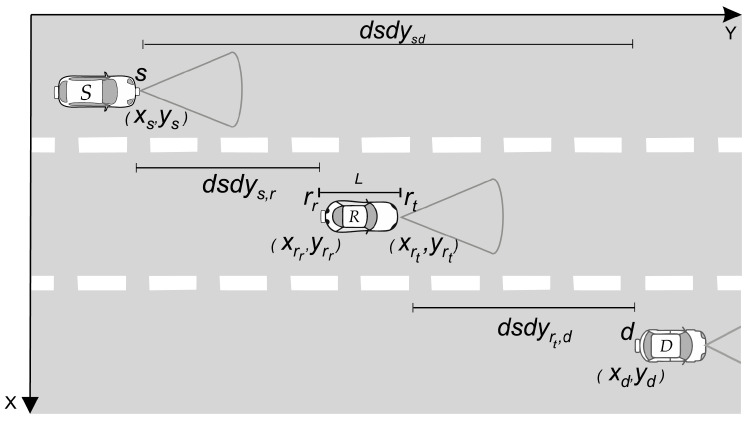
Schematic of a V2V-VLC cooperative network with an intermediate relay vehicle.

As schematically shown in Figure 1, the system consists of three cars; namely, the source (*S*), the destination (*D*) and an intermediary relay (*R*) vehicle, moving along a three-parallel-lane highway (running along the *y*-axis in Figure 1). The lanes are considered identical, having 3.5 m widths and centers at 1.75 m, 5.25 m and 8.75 m in relation to the *x*-axis in Figure 1.

**Figure 2:**
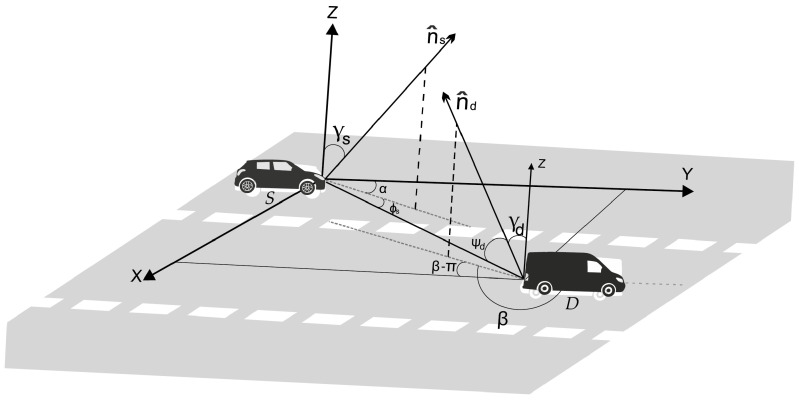
3D graphical representation of two cars *S* and *D* using V2V-VLC along a three-lane roadway. $\gamma_{s}$ and $\gamma_{d}$ represent the vertical tilt angles of the LED and PD, respectively, whereas $\phi_{s}$ corresponds to the irradiance angle with respect to ${\hat{n}}_{s}$. α and β denote the horizontal tilt angles with respect to ${\hat{n}}_{s}$ and ${\hat{n}}_{d}$, respectively. $\psi_{d}$ indicates the incidence angle with respect to ${\hat{n}}_{d}$. The unitary vectors ${\hat{n}}_{s}$ and ${\hat{n}}_{d}$ are used to denote the transmitter (LED) and receiver (PD) axes; i.e., they are normal to the corresponding surfaces.

For the sake of generality, we consider a three-dimensional (3D) model for the VLC link (LED-PD) in Figure 2. ${\hat{n}}_{s}$ and ${\hat{n}}_{d}$ are used to represent the unitary vectors normal to the transmitter (LED) and receiver (PD) axes, respectively, which, in general, can be slightly tilted by α ($\gamma_{s}$) and β ($\gamma_{d}$) with respect to the *y*-axis (*z*-axis), as depicted. The irradiance ($\phi_{s}$) and incident ($\psi_{d}$) angles were obtained from [13]

(1) $$\begin{array}{ccc} \phi_{s} & = & \arccos\left(\sin\gamma_{s}\cos\left[\alpha -\arctan\left(\frac{dsdx_{kl}}{dsdy_{kl}}\right)\right]\right), \end{array}$$

(2) $$\begin{array}{ccc} \psi_{d} & = & \arccos\left(-\sin\gamma_{d}\cos\left[\beta -\arctan\left(\frac{dsdx_{kl}}{dsdy_{kl}}\right)\right]\right), \end{array}$$

where $dsdy_{kl}$ and $dsdx_{kl}$ represent the horizontal and vertical distances between the transmitter and receiver. Subindices $k\in \left\{s,r_{t}\right\}$ and ($l\in \left\{r_{r},d\right\}$) are used to indicate whether the corresponding cars are working as source (*s*), destination (*d*) or as a relay in the transmitting ($r_{t}$) or receiving ($r_{r}$) mode, respectively.

The direct current gain, defined as [27]

(3) $$H_{kl}\left(0\right)=\left\{\begin{array}{cc} \frac{WA_{p}T}{d_{kl}^{2}}g\left(\psi_{d}\right)\cos\left(\psi_{d}\right) & \;,\;\;\;\;\;0\leq \psi_{d}\leq \psi_{c}, \end{array}\right.$$

will be used here to estimate the achievable signal-to-noise ratio (SNR) for a fixed transmit power. $d_{kl}=\sqrt{dsdy_{kl}^{2}+dsdx_{kl}^{2}}$ represents the distance between the transmitter and the receiver; $A_{p}$ is the area of the PD; *T* is the filter transmission coefficient; and *W* and $g(\psi_{d})$ are the radiant intensity of the emitting LED and the gain of the PD. $\psi_{c}<\pi /2$ is used for the aperture angle of the concentrator, also named the PD field-of-view (FOV). Considering the LED as an ideal Lambertian surface, the radiant intensity can be described by [27]

(4) $$W=\left[\frac{\left(m+1\right)}{2\pi }\right]\cos^{m}\left(\phi_{s}\right),$$

with $\;m=-\ln2/\ln\left[\cos\left(\phi_{1/2}\right)\right]$ indicating the order-index, where $\phi_{1/2}$ is the half-value angle of the LED. $g(\psi_{d})$, on the other hand, depends on the FOV and the PD refractive index (*n*) as [27]

(5) $$g\left(\psi_{d}\right)=\left\{\begin{array}{cc} \frac{n^{2}}{\sin^{2}\left(\psi_{c}\right)} & ,\;0\leq \psi_{d}\leq \psi_{c},\\ 0 & ,\;\psi_{d}\geq \psi_{c}. \end{array}\right.$$

### 2.2. Curved Roadway Scenario

**Figure 3:**
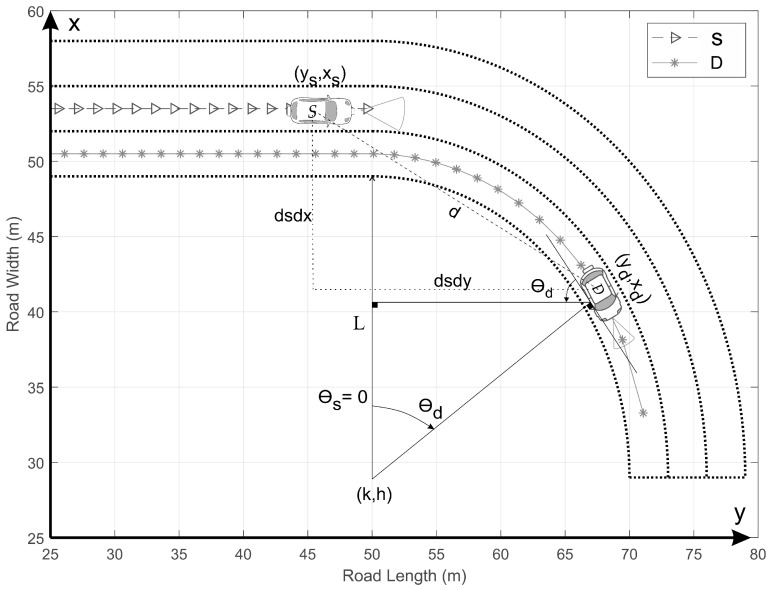
Schematic of two cars using V2V-VLC along a curved roadway. $\theta_{s}$ and $\theta_{d}$ represent the rotation of the LED- and PD-axis with respect to the *x*-axis, respectively. *L* denotes the internal radius of the semicircular roadway section. dsdx and dsdy correspond to the differential distances between *S* and *D* along the *x* and *y* axes.

The idea in this section is to extend the previous modeling to the case of V2V-VLC in the presence of curved roads. In contrast to the previous section, where the LED and PD axes were fixed parallel to the *y*-axis, i.e., α=0 and β=π (see Figure 2), we must now consider them to be rotating around the *x*-axis when traveling along a curved roadway. Rotation angles are measured with respect to the *x*-axis, as illustrated in Figure 3, and labeled as $\theta_{s}$ and $\theta_{d}$ for the LED and PD axes; i.e., $\alpha =\theta_{s}$ and $\beta =\pi -\theta_{d}$. L=20 m is the radius of the internal border of the semicircular roadway, as depicted. The coordinate (k,h) [(y,x)] is used to represent the center of the semicircular section, where *k* = 29 m and *h* = 50 m.

**Figure 4:**
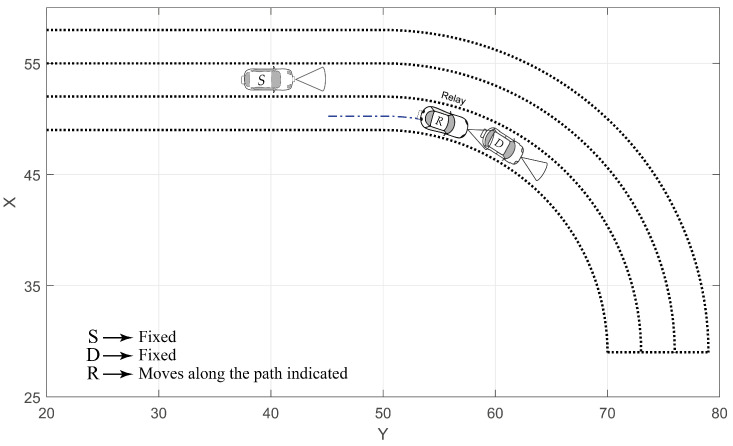
Schematic of the cooperative communication along a curvilinear roadway. *D* is considered fixed at different angular positions, while *R* follows the dash-dotted path between *S* and *D*. For all cases *S* is considered as having $y_{s}$ < 50 m.

For comparison purposes, we will consider both the cooperative and non-cooperative communication mechanisms. In the non-cooperative communication we used the scenario represented in Figure 3, whereas for the cooperative one we considered the scenario illustrated in Figure 4. In the cooperative scenario, we use the car *R* moving along the same lane of the car *D*, as depicted in Figure 4. For simplicity, all the calculations were made considering $\theta_{s}=0$.

Our study of the V2V-VLC in this section is limited to the scenarios represented in Figure 3 and Figure 4, i.e., for $y_{s}\leq h$. The corresponding vehicle lengths are considered as $l_{s}=l_{d}=5$ m. The geometrical analysis of Figure 3 can be divided into two different situations. First, for $y_{s}\leq \left(h-dsdy_{sd}\right)$, i.e., $y_{d}\leq h$, the V2V-VLC occurs along a rectilinear roadway ($\theta_{s}=\theta_{d}=0$), analogously to the previous section. Second, for $\left(h-dsdy_{sd}\right)<y_{s}\leq h$, S continues traveling along a rectilinear path ($\theta_{S}=0$), whereas D enters into the semicircular roadway section ($\theta_{d}>0$). Considering the LEDs and PD located on the fronts and rears of the cars, respectively, and *y* measured with respect to the center of each car, we found

(6) $$\begin{array}{ccc} dsdy_{sd} & = & y_{d}-y_{s}-\frac{l_{d}}{2}\cos\theta_{d}-\frac{l_{s}}{2}, \end{array}$$

(7) $$\begin{array}{ccc} dsdx_{sd} & = & x_{d}-x_{s}-\frac{l_{d}}{2}\sin\theta_{d}, \end{array}$$

(8) $$\begin{array}{ccc} \phi_{s} & = & \arccos\left(\sin\gamma_{s}\cos\left[\alpha -\arctan\left(\frac{dsdx_{sd}}{dsdy_{sd}}\right)\right]\right), \end{array}$$

(9) $$\begin{array}{ccc} \psi_{d} & = & \arccos\left(-\sin\gamma_{d}\cos\left[\beta -\arctan\left(\frac{dsdx_{sd}}{dsdy_{sd}}\right)\right]\right), \end{array}$$

where $\beta =\pi -\theta_{d}$. To avoid using vehicle speeds, we analyze the V2V-VLC perfomance using a constant value for the difference $y_{d}-y_{s}=L$. This constraint is used to meet the limiting condition ${\hat{n}}_{s}⊥{\hat{n}}_{d}$; i.e., α=0 and β=π/2, when D reaches the end of the semicircular roadway in Figure 3. Thus, we found that the PD axis rotation can be easily written as $\theta_{d}=\tan^{-1}\left[\frac{y_{s}+L-h}{\sqrt{L^{2}-{\left(y_{s}+L-h\right)}^{2}}}\right]$ when moving along the curved road. These geometrical analyses are directly extended to the cooperative communication case by using the relays in the receiving and transmitting mode as the destination and source vehicle, respectively.

## 3. BER Analysis

In this section, we introduce the analysis of the cooperative FD V2V-VLC protocol. Self-interference is neglected for FD V2V-VLC, differently to the RF transmission, as the LED and PD are isolated. Hence, the received signal at node *l* of the signal from *k* can be expressed as:

(10) $$\begin{array}{ccc} v_{kl} & = & \zeta P_{k}H_{kl}\left(0\right)u_{k}+N_{kl}, \end{array}$$

where $P_{k}$ and $u_{k}$ are the power and the message sent by the corresponding transmitter, respectively. $N_{kl}$ represents the Gaussian additive noise at the node *l*, with variance $\sigma^{2}$, and ζ is the responsivity (at a fixed wavelength) of the photodiode expressed in A/W.

The signal to noise ratio (SNR) is calculated for the channel k→l as:

(11) $$\begin{array}{c} {\mathrm{SNR}}_{kl}=\frac{{\left[\zeta P_{k}H_{kl}\left(0\right)\right]}^{2}}{\sigma^{2}}, \end{array}$$

with $\sigma^{2}=\sigma_{\mathrm{shot}}^{2}+\sigma_{\mathrm{thermal}}^{2}$ representing the noise variance; i.e., the sum of the shot noise and the thermal noise variances. The shot noise variance is calculated by

(12) $$\sigma_{\mathrm{shot}}^{2}=2q\zeta P_{k}H_{kl}\left(0\right)B+2q\zeta P_{bg}I_{2}B,$$

where *q* represents the electron charge, *B* is the considered bandwidth, $P_{bg}$ is the background noise power and $I_{2}$ is the noise bandwidth factor of the background noise. The thermal noise is generated within the transimpedance receiver circuitry [28] and its variance ($\sigma_{\mathrm{thermal}}^{2}$) is expressed by:

(13) $$\sigma_{\mathrm{thermal}}^{2}=\left(\frac{8\pi K_{b}T_{A}}{G}\right)\eta A_{p}I_{2}B^{2}+\left(\frac{16\pi^{2}K_{b}T_{A}\Gamma }{g_{m}}\right)\eta^{2}A_{p}I_{3}B^{3},$$

where $K_{b}$ is the Boltzman constant, $T_{A}$ is the absolute temperature, *G* is the voltage gain in open loop, η is the capacitance per unit area of the photodetector, Γ is the noise factor of the FET (field-effect transistor) channel, $g_{m}$ is the FET transconductance and $I_{3}$ is the noise bandwidth factor. The modulation used in this transmission is on-off-keying (OOK), as it is proposed in the IEEE 802.15.7 standard for VLC communication [29,30]. The BER for each link is calculated as [31]

(14) $$\begin{array}{ccc} {\mathrm{BER}}_{sr_{r}} & = & Q(\sqrt{{\mathrm{SNR}}_{sr_{r}}}), \end{array}$$

(15) $$\begin{array}{ccc} {\mathrm{BER}}_{r_{t}d} & = & Q(\sqrt{{\mathrm{SNR}}_{r_{t}d}}), \end{array}$$

where the Q(·) function

(16) $$Q\left(x\right)=\frac{1}{\sqrt{2\pi }}\int_{x}^{\infty }e^{\frac{-a^{2}}{2}}da,$$

represents the probability of a normal (Gaussian) random variable having a value grater than *x* standard deviations.

The overall error performance of the dual-hop cooperative communication scheme, considering the intermediary node *r*, is then given by

(17) $${\mathrm{BER}}_{\mathrm{coop}}=1-\left(1-{\mathrm{BER}}_{sr_{r}}\right)\left(1-{\mathrm{BER}}_{r_{t}d}\right).$$

When direct transmission is possible, e.g., there is a LOS between *S* and *D* cars, the overall error performance of the non-cooperative scheme can be written as

(18) $$\begin{array}{ccc} {\mathrm{BER}}_{sd} & = & Q(\sqrt{{\mathrm{SNR}}_{sd}}). \end{array}$$

## 4. Numerical Results and Discussions

This section presents a numerical study of the performance for the proposed V2V-VLC cooperative communication scheme. We used the parameters in Table 1 for all the simulations in this work, according to [13,32]. The *S* vehicle was also considered to be transmitting beacons of length of 300 bytes (N = 2400 bits) [33] to obtain the numerical results.

### 4.1. Straight Road Scenario

**Figure 5:**
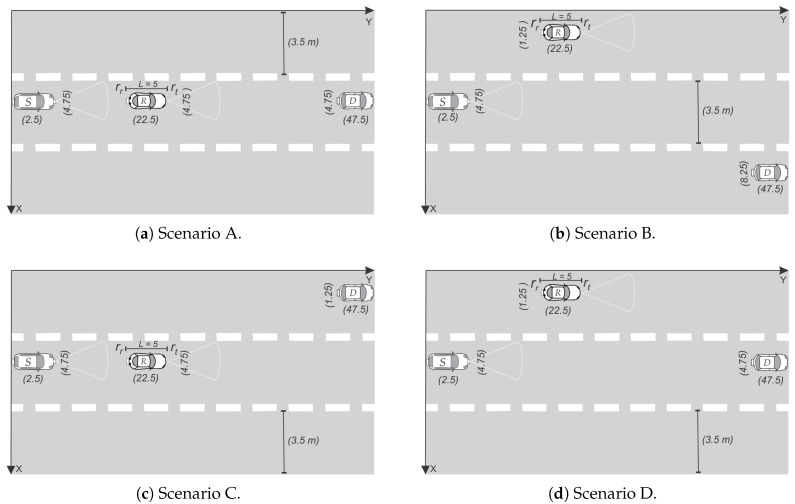
Pictorial representation of the four different scenarios of simulation for the straight roadway case.

In order to study the cooperative BER for different straight roadway scenarios, we evaluate four scenarios labeled as Scenarios A, B, C and D in Figure 5. For comparative purposes, we considered the same center-to-center horizontal distance (45 m) between the source and destination vehicles in all the scenarios, whereas the relay moves in between *S* and *D* with a minimum horizontal separation of 2 m from each. All cars are considered following rectilinear trajectories.

**Figure 6:**
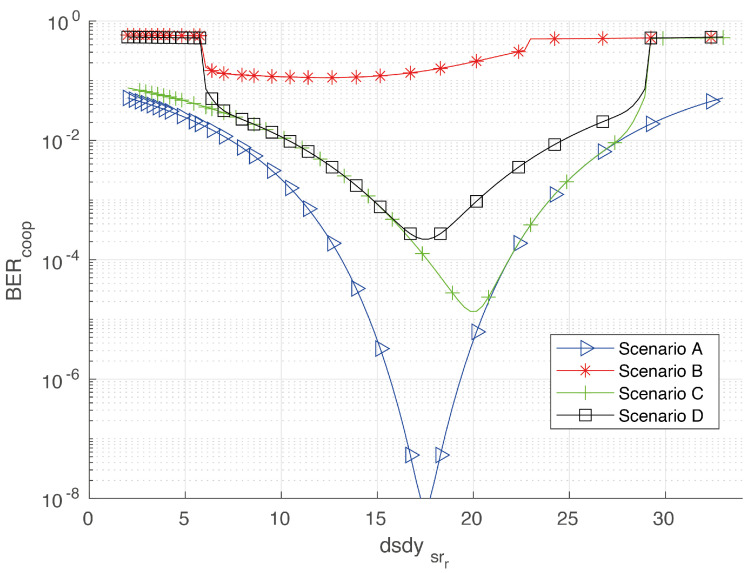
Cooperative bit error rate (BER) for different scenarios. Calculations were made varying $dsdy_{sr_{r}}$ between *S* and *D*, which were considered 40 m apart from each other.

**Figure 7:**
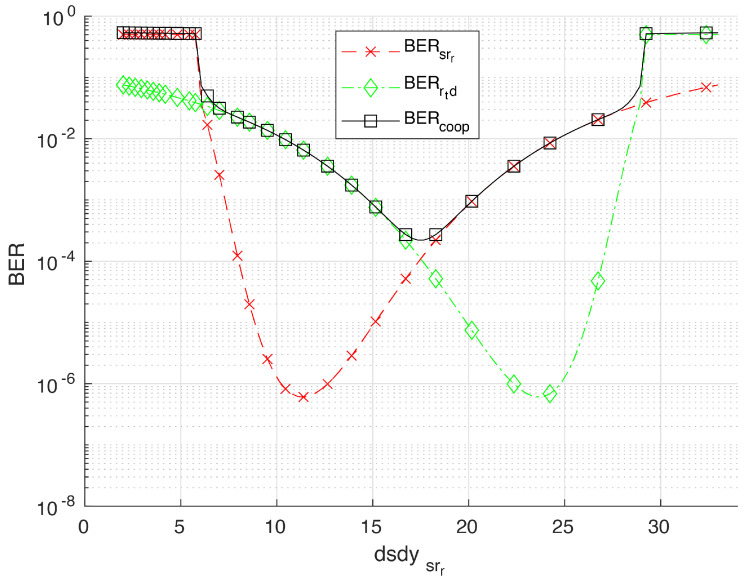
Results for the cooperative BER associated with the scenario D in Figure 5d, considering an intermediate relay.

Let us begin discussing the most simple case represented in Figure 5a; i.e., all cars are moving along the central lane. As the FOV is guaranteed for this rectilinear arrangement, this is also the best scenario. Numerical results for the cooperative BER for this case are presented by blue triangle-line in Figure 6. An optimum cooperative BER is found for a source-relay distance of 17.5 m, which represents the case where the center of the relay is half the distance between *S* and *D*. From Figure 6, we also note that the cooperative BER tends to get worse for the scenarios B, C and D. In particular, the scenario B (Figure 5b) has the worst performance, as the relay is very far from the destination and cannot help the source in the absence of a LOS channel. For a better explanation of the cooperative communication in this section, we extend the analysis of scenario D in Figure 7. From this last figure, it can be noted that the optimum value of the cooperative BER occurs when ${\mathrm{BER}}_{sr_{r}}={\mathrm{BER}}_{r_{t}d}$, which represents the case where the center of the relay is half the distance between *S* and *D*. Such symmetrical behavior noted for ${\mathrm{BER}}_{sr_{r}}$ and ${\mathrm{BER}}_{r_{t}d}$ is because the source and destination are traveling along the same lane. As the relay *R* starts moving close to the source (*S*), this $S\Rightarrow R(r_{r})$ communication is almost outside the FOV of $R(r_{r})$, which explains the large BER values despite the small $dsdy_{s,r_{r}}$ distance. The same analysis applies for the symmetrical $R(r_{t})\Rightarrow D$ communication link.

### 4.2. Curved Road Scenario (Non-Cooperative Communication)

**Figure 8:**
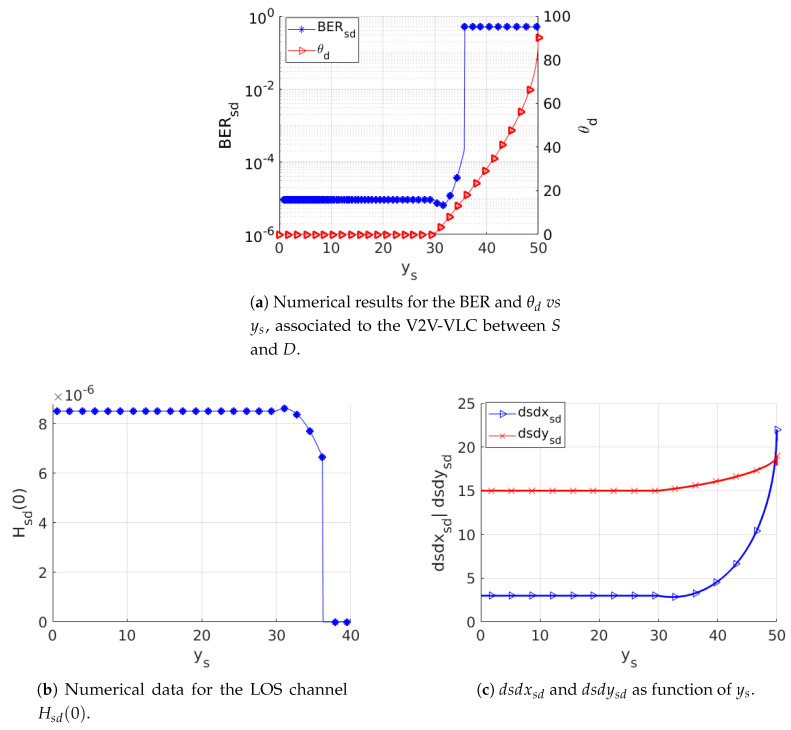
Performance analysis of the non-cooperative V2V-VLC along a curved roadway scenario.

We will now discuss the BER for the vehicles communicating along the curved roadway scenario represented in Figure 3. As previously mentioned, the PD-axis rotates around the *x*-axis as D moves along the curved roadway section. In Figure 8a, we present the numerical results for the BER and the corresponding $\theta_{d}$ as function of $y_{s}$. We may note a constant BER = 10^−5^ for $y_{s}$ ≤ 30 m, which corresponds to the straight roadway section $\theta_{d}$ = 0. For $y_{s}$ > 30 m, 0° < $\theta_{d}$ ≤ 90°, we note a rapidly increasing of the BER associated to a diminishing in the corresponding FOV at *D*. We can also note, from this figure, that there is a threshold $\theta_{d}$= 16.8° ($y_{s}$ = 36.2 m) above which the communication is disrupted; i.e., the BER becomes 0.5. The corresponding channel disruption is presented in Figure 8b, where $H_{s,d}\left(0\right)$ is presented for V2V-VLC between *S* and *D*. Figure 8c presents the corresponding $dsdy_{s,d}$ and $dsdx_{s,d}$ distances as functions of $y_{s}$, from which we may only note a slight change, making evident that the communication disruption is completely due to the loss of FOV between *S* and *D*.

### 4.3. Cooperative Communication in the Curved Roadway Scenario

**Figure 9:**
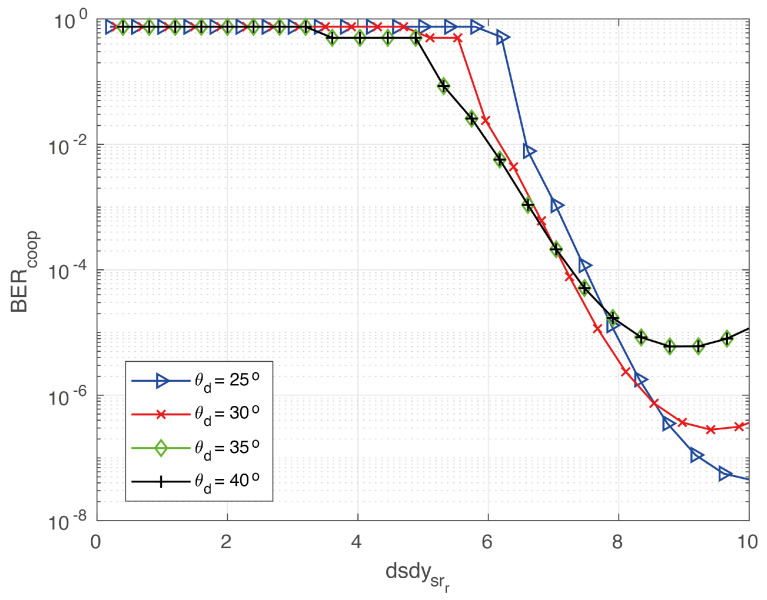
Cooperative BER as a function of $dsdy_{sr_{r}}$ for different values of $\theta_{d}$.

In the previous section, for non-cooperative V2V-VLC, we found that the communication becomes disrupted for angles as small as $\theta_{d}$ > 16.8° ($\theta_{s}$ = 0°). Here, we show that a cooperative V2V-VLC can be used to reach higher $\theta_{d}$ values. In doing so, we considered three cars named *S*, *R* and *D* moving along the roadway illustrated in Figure 4. As noted from the FOV analysis in Figure 6, the BER results exhibit acceptable values for cases where R and D, or S and R, travel along the same lane. In the curved scenario, the FOV changes dramatically in comparison to the rectilinear scenario. Thus, the analyses are limited to the scenario in Figure 4 in order to study the detrimental effects on the BER due to the curvilinear lanes. For simplicity in the calculations, *S* and *D* were taken fixed at different angular positions, whilst *R* was used as moving between them. In particular, results were calculated for *D* placed at $\theta_{d}$ = 25°, 30°, 35° and 40°, as schematized in Figure 4. Results associated to the cooperative BER for these $\theta_{d}$ values are presented in Figure 9, from where we directly note that the communication link can be extended to angles up to 40° exhibiting good communication performances. The coordinates of the source and destination used for calculations in Figure 9, for different $\theta_{d}$, are given by the Table 2. These results indicate that the cooperative V2V-VLC protocols constitute the most successful way to avoid communication disruptions for cars communicating along realistic curvilinear roadway scenarios. Furthermore, in the case of low-density-car highway environments, our results can be extended to properly use the highway VLC infrastructure in order to avoid communication disruptions.

## 5. Conclusions

Summarizing, we have numerically analyzed the use of full-duplex cooperative VLC considering a vehicular network composed by cars named source, relay and destination. The analyses were made considering rectilinear and curvilinear highway scenarios. For comparative purposes, we also considered direct communication between the source and destination. The cars were also considered at different lanes and positions along the highway to analyze the corresponding BERs. From the results in this work, we concluded that even for short distances and direct communication, VLC can be disrupted because the loss of LOS between the LED and PD; i.e., there is a minimum distance that allows the FOV requirement for the VLC link. Moreover, the best BER values are reached when the relay and destination, or the source and relay, are traveling along the same lane. Significantly, in the case of curved roadway scenarios, we found that the cooperative full-duplex communication protocol can be used to extend the reach of VLC link to angles around 40° between the LED and PD, in contrast to ∼17° in direct communication. 

## Figures and Tables

**Table 1:** System parameters.

Parameter	Symbol	Value
FOV of the receiver	$\psi_{c}$	π/6 rad
Half value angle of an LED	$\phi_{1/2}$	π/12 rad
Internal refractive index	*n*	1.5
Area of incidence at receiver	$A_{p}$	1 cm^2^
Filter Transmission Coefficient	*T*	1
Detector Responsivity	ζ	0.56 A/W
Ambient Temperature	$T_{A}$	300 K
Open loop channel gain	*G*	10
FET Transconductance	$g_{m}$	30 mS
Fixed PD Capacitance/area	η	112 pF/cm^2^
Noise Bandwidth Factor	$I_{2},I_{3}$	0.562, 0.0868
Background Noise Power	$P_{bg}$	16 dBm
LED Power	$P_{k}$	0.3 W
Horizontal Inclination angle	α	0 rad
Horizontal Inclination angle	β	π rad
Vertical Inclination angle	$\gamma_{1},\gamma_{2}$	π/2 rad
Code Rate	CR	20 Mbps
Electronic Charge	*q*	$1.6021\times 10^{-19}$ C
FET Channel noise factor	Γ	1.5
Boltzmann Constant	$K_{b}$	$1.3806\times 10^{-23}$ J/K
System Bandwidth	*B*	20 MHz
Number of bits	N	2400 bits

**Table 2:** Coordinates of sources and destinations for different values of $\theta_{d}$.

Node Positions				
$\theta_{d}$	$x_{s}$	$y_{s}$	$x_{d}$	$y_{d}$
25°	53.75 m	38.50 m	48.23 m	59.03 m
30°	53.75 m	40.00 m	47.40 m	60.62 m
35°	53.75 m	41.50 m	46.38 m	62.22 m
40°	53.75 m	42.90 m	45.24 m	63.71 m
